# Association of Antihypertensive Drug-Related Gene Polymorphisms with Stroke in the Chinese Hypertensive Population

**DOI:** 10.1155/2024/5528787

**Published:** 2024-01-23

**Authors:** Huixia Liu, Hua Zhong, Ying Lin, Linzhi Han, Mengshi Chen, Tao Tang, Jing Deng

**Affiliations:** ^1^Xiangya School of Public Health, Central South University, Changsha, China; ^2^Department of Cardiology, Xiangya Hospital, Central South University, Changsha 410008, China; ^3^Department of Clinical Pharmacology, Xiangya Hospital, Central South University, 87 Xiangya Road, Changsha 410008, China; ^4^Hunan Provincial Key Laboratory of Clinical Epidemiology, Central South University, Changsha, China; ^5^Tongxiang Center for Disease Control and Prevention, Tongxiang, Zhejiang 314500, China

## Abstract

**Background:**

Antihypertensive therapy is crucial for preventing stroke in hypertensive patients. However, the efficacy of antihypertensive therapy varies across individuals, partially due to therapy-related genetic variations among individuals. We investigated the association of antihypertensive drug-related gene polymorphism with stroke in patients with hypertension.

**Methods:**

Demographic information, medication, and outcome data were obtained from a hypertensive patient management system, and a PCR fluorescence probe technique was used to detect 7 gene polymorphic loci (*CYP2D6∗10*, *ADRB1*, *CYP2C9∗3*, *AGTR1*, *ACE*, *CYP3A5∗3*, and *NPPA*), and these loci were compared between patients with and without stroke. Logistic regression was performed to analyze the association of these genetic variations with stroke risk in hypertensive patients while controlling for potential factors.

**Results:**

The prevalence of stroke in the hypertensive population in Changsha County of Hunan Province was 2.75%. The mutation frequencies of *ADRB1* (*1165G* > *C*), *CYP2D6∗10*, *CYP2C9∗3*, *AGTR1* (*1166A* > *C*), *ACE* (*I*/*D*), *NPPA* (*2238T* > *C*), and *CYP3A5∗3* were 74.43%, 57.23%, 4.26%, 5.71%, 31.62%, 1.17%, and 69.58%, respectively. Univariate analysis revealed that *ADRB1* polymorphism was associated with stroke (*χ*^2^ = 8.659, *P* < 0.05), with a higher stroke risk in the *CC* group than in the *GC* and *GG* groups (*GC* + *GG*). Multivariate unconditional logistic regression analysis showed that the *CC* genotype in *ADRB1* (vs. the *GC* + *GG* genotype) was associated with an increased risk of stroke [odds ratio (OR) = 1.184, *P*<0.05] in hypertensive patients. No association was observed between *CYP2D6∗10*, *CYP2C9∗3*, *AGTR1* (*1166A* > *C*), *ACE* (*I*/*D*), *CYP3A5∗3*, and *NPPA* (*2238T* > *C*) polymorphisms and stroke.

**Conclusions:**

*ADRB1* (*1165G* > *C*) gene polymorphism is associated with the risk of stroke in Chinese hypertensive patients. The *CC* genotype is correlated with a higher risk of stroke than the *GC* + *GG* genotype.

## 1. Introduction

Stroke, which is the second leading cause of death globally, is a chronic noninfectious disease hampering public health [1]. Compared to that in 1990, the absolute number of stroke events worldwide increased by 70.0% in 2019, and the number of deaths increased by 40%. Currently, stroke is the leading cause of death and disability among adults in China; its morbidity, mortality, and number of cases are the highest worldwide [2, 3].

Hypertension is the most important and modifiable high-risk factor for stroke development [4–6]. The World Health Organization (WHO) estimates that approximately 54% of all stroke cases worldwide can be attributed to hypertension [7]. Hypertension control could significantly reduce the incidence of stroke [8]. The drugs for the treatment of hypertension are mainly divided into five categories: beta receptor blockers (BBs), angiotensin-II receptor antagonists (ARBs), angiotensin-converting enzyme inhibitors (ACEIs), calcium channel blockers (CCBs), and diuretics. However, community attainment of hypertension treatment remains dismal, with less than a quarter being controlled [9, 10]. Uncontrolled hypertension may significantly contribute to high mortality from stroke [11].

Hypertension is a polygenic disease, and differences in antihypertensive efficacy are partially associated with genetic variations among individuals [12, 13]. A previous study summarized the pharmacogenomics of hypertension treatment, and the most common genetic variants involved in the metabolism of the above five classes of drugs included *ADRB1* (*1165G* > *C*), *CYP2D6∗10*, *CYP2C9∗3*, *AGTR1* (*1166A* > *C*), *ACE* (*I*/*D*), *CYP3A5∗3*, and *NPPA* (*2238T* > *C*) [14].

Differences in these gene polymorphisms in response to antihypertensive therapy have been described previously in the literature. Certain genotypes exhibit a greater reduction in blood pressure (BP) and/or long-term benefit with antihypertensive drug use [15]. In particular, the *CC* genotype of *ADRB1* (*1165G* > *C*), *DD* genotype of *ACE* (*I*/*D*), *C* allele carrier of *AGTR1* (*1166A* > *C*), and *NPPA* (*2238T* > *C*) are reported to be associated with a significant reduction in BP [16–19]. Mutations in cytochrome P450 enzyme family members, such as *CYP2D6*, *CYP2C9*, and *CYP3A5*, mainly affect the activities of metabolic enzymes. Patients using antihypertensive drugs and having an increased active *CYP* phenotype may have increased metabolic capacity; in contrast, those with an inactive *CYP* phenotype may display increased clinical effects and bradycardia following the use of metoprolol and other antihypertensive drugs [20]. A previous study reported that the *CYP2C9∗3* allele may be associated with a decrease in the activity of the *CYP2C9* enzyme in the Asian population [21], while *CYP3A5∗3* is correlated with effective control of BP with amlodipine (CCB) in hypertensive patients following renal transplantation via decreasing the activity of the *CYP3A5* enzyme [22]. The association of the *CYP2D6* mutation with the outcome of antihypertensive therapy with BBs is controversial. *CYP2D6∗10* significantly affected the pharmacokinetics of metoprolol in the Chinese population [23]; however, no significant effect on the outcome of carvedilol and propranolol therapy was observed [24, 25].

*ADRB1* (*1165G* > *C*), *CYP2D6∗10*, *CYP2C9∗3*, *AGTR1* (*1166A* > *C*), *ACE* (*I*/*D*), *CYP3A5∗3*, and *NPPA* (*2238T* > *C*) are associated not only with BP reduction but also with disease prognosis. A few previous studies revealed that a mutation in *ADRB1* (*1165G* > *C*) was associated with the survival of patients with heart failure by increasing the dose of BBs [26]. In patients with chronic heart failure and the *ADRB1 CC* genotype, the use of BBs also appears to reduce the risk for cardiovascular events [26, 27]. However, to the best of our knowledge, no studies have focused on the relationship between all above-mentioned genes and stroke. A community registry of hypertensive patients was started in Changsha County, Hunan Province, in 2013; this system registers the BP treatment and outcomes of hypertensive patients through follow-ups. In 2017, a test of the seven genes polymorphisms mentioned above was conducted in hypertensive patients. Our study aimed to explore the association between different antihypertensive-related genotypes and stroke. It would provide some evidence for preferential selection of antihypertensive medication in the hypertensive population in Changsha County, Hunan Province.

## 2. Methods

### 2.1. Study Population

The study population was selected from hypertensive patients registered in the hypertension management system at each township health center and street health service center in Changsha County, Hunan Province, between January 1, 2013, and January 1, 2018.

Inclusion criteria for study participants: all the hypertensive patients registered in the management system; these patients were enrolled based on the criteria of the Chinese Guidelines for the Prevention and Treatment of Hypertension (2010 Revision) [28]. (1) participants had a systolic blood pressure (SBP) ≥ 140 mmHg (1 mmHg = 0.133 kPa) or a diastolic blood pressure (DBP) ≥ 90 mmHg three times on different days; (2) participants were previously diagnosed with hypertension; and (3) participants were currently taking BP medications. Exclusion criteria for study participants: (1) participants registered in the system but with no record of BP; (2) participants without any follow-up records; and (3) participants under the age of 18 years.

### 2.2. Data Source

All patient data were obtained from the same above-mentioned hypertension management system. This system is a part of the National Basic Public Health Service Project Management Information System, which records the medical information of all registered and follow-up hypertensive patients. According to the basic public health service guidelines, hypertensive patients were followed up face-to-face four times a year, and they had to undergo physical examinations yearly. Family doctors and public health personnel in the community health service centers were responsible for registering, following up, and managing patients with hypertension within their jurisdiction. The Centers for Disease Control and Prevention were responsible for developing technical programs, personnel training, and quality control of follow-up data. All medical follow-up information of the patients must be faithfully recorded in the system database by the physicians monitoring the patients. Strokes and other illnesses in follow-up patients must be documented in detail; stroke and other cardiovascular events must be diagnosed and confirmed by a high-level hospital.

This study was approved by the Ethics Committee of Xiangya School of Public Health, Central South University (approval number: XYGW-2022-73), and informed consent was taken from all individual participants.

### 2.3. Data Collection

Demographic information, medication, and outcome data were derived from the basic patient information module, physical examination information module, drug exposure module, and follow-up module in the hypertension patient management system in Changsha County, Hunan Province.

#### 2.3.1. Basic Patient Information Module

Data exported from this module included participants' ID number, date of birth, sex, type of usual residence, education level, marital status, presence of coronary heart disease at the time of first registration, presence of diabetes at the time of first registration, presence of chronic obstructive pulmonary disease (COPD) at the time of first registration, presence of tumor at the time of first registration, family history of stroke, family history of hypertension, and family history of diabetes.

#### 2.3.2. Physical Examination Module

Annual height and weight checks and positive physical examination results were exported from this module.

#### 2.3.3. Follow-Up and Medication Modules

Data exported from these modules included the use and compliance of antihypertensive drugs, the drug names (trade names), usage times, and self-reported adverse reactions.

#### 2.3.4. Genetic Polymorphism Data

Between January 1, 2017, and December 31, 2017, oral mucosa-shedding cells of hypertensive patients were collected using oral mucosa swabs during the annual physical examination conducted by community health services centers of Changsha County. And genomic DNA was extracted using a commercial nucleic acid separation kit (Promega, USA) and frozen at −80°C. PCR fluorescence probe technique was used to detect *ADRB1* (*1165G* > *C*), *CYP2D6∗10*, *CYP2C9∗3*, *AGTR1* (*1166A* > *C*), *ACE* (*I*/*D*), *CYP3A5∗3*, and *NPPA* (*2238T* > *C*) polymorphisms (hypertension drug-related gene chip detection kit, sequencing primer, and other reaction fluids are products of Hunan Honghao Biopharma Co., Ltd., China). The PCR conditions are as follows: predenaturation at 95°C for 2 min; denaturing 40 s at 95°C; annealing 40 s at 60°C; extending 40 s at 72°C; a total of 40 cycles; and the final extension step at 72°C for 5 min. GenePix4 100A scanner was used to conduct quantitative analysis of the scan results with its own system, GenePix6.0, and the analysis software was used to interpret the genotype according to the set Cuff value. For genes and related polymorphisms, rs number, and interested genotypes, refer to the supplementary documents (Table S1).

### 2.4. Data Cleaning and Merging

#### 2.4.1. Data Cleaning

This study included data derived from the basic information module with one record for each participant and from the physical examination follow-up and medication modules, generating multiple records for each participant. Therefore, we carried out data cleaning according to the following principles:

1. For the physical examination module, height, weight, BP, and positive physical examination results (such as stroke, coronary heart disease, and kidney disease) were mainly extracted.
2. For follow-up and medication modules, the inpatient and outpatient diagnoses, the patient's main antihypertensive medication, and the duration of medication use were extracted. The antihypertensive drugs were classified into five categories: BBs, ARBs, ACEIs, CCBs, and diuretics. CCBs include dihydropyridine and nondihydropyridine drugs. Accumulated days of each antihypertensive drug taken by the patients were calculated according to the medication status with and without specific medicine, and compliance was recorded at each follow-up visit. If patients complied well with antihypertensive drugs, the duration of drug exposure was considered as 120 days during one follow-up period based on 4-month visit intervals. In the case of poor compliance of patients with drugs, the number of days of drug use during the follow-up period was registered or calculated by the time between the start and discontinuation of drug use.
3. Poststroke medication exposure was not analyzed.

#### 2.4.2. Data Merge

Basic information, BP at each examination and visit, antihypertensive drug use, stroke, and other conditions were combined with “resident ID card” as the unique identification number and name for the checking information.

### 2.5. Research Variable Determination

#### 2.5.1. Stroke Definition

In this study, stroke was defined as follow-up and physical examination records containing “stroke,” “cerebral hemorrhage,” “cerebral infarction,” or records with diagnosis codes (ICD-10) containing I60–I64. The cases extracted from the follow-up records require the appropriate diagnostic basis (inpatient treatment in hospitals at or above the county level or with a high-resolution imaging diagnosis). If a “stroke” record with unique identity was detected in the follow-up or physical examination record, it will be recorded as one patient. If “stroke” records with the same identity were detected both in the follow-up and physical examination records, they will be recorded as one patient only.

#### 2.5.2. Body Mass Index Classification

Body mass index (BMI) was calculated based on the height and weight of patients at their earliest registration dates and was divided into three categories: <18.5, 18.5∼24, and >24 kg/m^2^.

#### 2.5.3. Age Classification

According to the age at the earliest registration dates in the system, participants were divided into three age groups: 18–45, 45–60, and >60 years.

#### 2.5.4. Relevant Family History and Disease Incidence

Family history was defined as the patient's immediate family members with hypertension, stroke, diabetes, or other diseases. Presence of coronary heart disease, COPD, diabetes, or tumor refers to the occurrence of these diseases in the patients; the related data were generally obtained from the registration inquiry information belonging to the basic information data.

#### 2.5.5. Control of SBP and DBP

According to the hypertension prevention and treatment guidelines, control of SBP and DBP is defined as follows: (1) general hypertension patients with BP of <140/90 mmHg, (2) hypertension comorbid with diabetes or heart failure with BP of <130/80 mmHg, (3) hypertension comorbid with coronary heart disease or chronic kidney disease with BP of <140/90 mmHg, and (4) older patients aged >65 years with BP of <150/90 mmHg. If the above criteria were met, BP was considered to be under control; otherwise, it was not controlled.

#### 2.5.6. History of Drug Exposure

According to the WHO [29] and Chinese guidelines [30], if the antihypertensive effect is not good or does not achieve the goal level after 4 weeks of initial treatment, a combination of medications or a change of medication is required. Therefore, we defined a history of use of an antihypertensive drug as the drug being used for more than 28 days.

Administering antihypertensive drugs (BBs, ARBs, ACEIs, CCBs, and diuretics) refers to a patient's taking a specific antihypertensive drug for several days. According to Guidelines for Rational Drug Use of Hypertension in China (Second Edition) [30], the use of BBs, ARBs, ACEIs, CCBs, and diuretics should be changed or used in combination therapies after four weeks of administration. Therefore, we defined the exposure time of BBs, ARBs, ACEIs, CCBs, or diuretics as more than 4 weeks.

### 2.6. Statistical Analysis

SPSS 22.0 software and R Studio were used for data analyses. Quantitative data and categorical data were expressed as mean and standard deviation (SD) and as frequency or percentage, respectively. A chi-square test was performed to compare the genotypes and allele frequency and detect Hardy–Weinberg equilibrium (HWE). Logistic regression was used to determine the association of the polymorphisms of each antihypertensive drug-related gene with stroke, considering the possible confounding factors, including general demographic information, the presence of other main diseases, family history of disease, and antihypertensive drug use. All hypothesis tests were two-tailed, and *P* < 0.05 was considered statistically significant. A further subgroup analysis was performed, considering the clinical heterogeneity of hypertension patients.

## 3. Results

### 3.1. General Information

**Figure 1:**
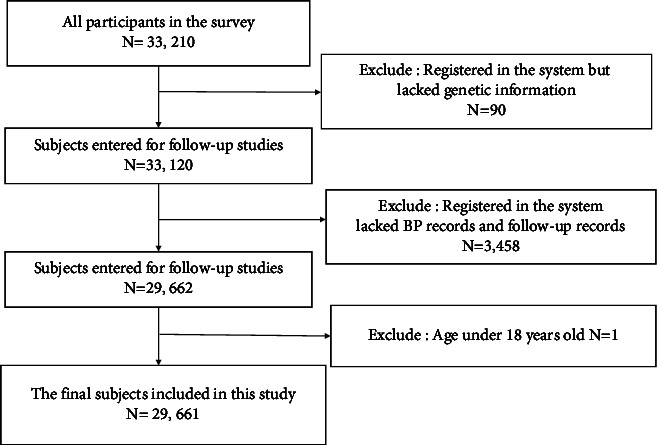
Flowchart of participants.

A total of 33,210 participants were registered in the system. We excluded 90 patients who were enrolled in the system but lacked genetic information and 3,458 patients who lacked BP records and follow-up records. One patient under 18 years was excluded. Finally, 29,661 patients with hypertension were included in the follow-up analysis (Figure 1); among them, 816 (2.75%) had stroke. Significant differences were observed between the stroke patients and the nonstroke patients in terms of sex, age, BMI, educational level, presence of coronary heart disease, presence of COPD, presence of tumor, family history of stroke, family history of hypertension, and the use of ACEIs, diuretics, or CCBs (*P* < 0.05). In contrast, no significant differences were observed between these two groups in terms of resident type, marital status, control of SBP, control of DBP, presence of diabetes, family history of diabetes, ARBs exposure, and BBs exposure (*P* > 0.05) (Table 1).

### 3.2. Distribution of Drug-Related Genes in Hypertensive Patients and Their Relationship with Stroke

The mutation frequencies of *ADRB1* (*1165G* > *C*), *CYP2D6∗10*, *CYP2C9∗3*, *AGTR1* (*1166A* > *C*), *ACE* (*I*/*D*), *NPPA* (*2238T* > *C*), and *CYP3A5∗3* were 74.43%, 57.23%, 4.26%, 5.71%, 31.62%, 1.17%, and 69.58%, respectively (Table 2). Hardy–Weinberg balance test was conducted on the distribution of antihypertensive drug-related genotypes in the control group, and no significant difference was observed in *ADRB1* (*1165G* > *C*), *CYP2C9∗3*, *ACE* (*I*/*D*), and *CYP3A5∗3* polymorphism loci (*P* > 0.05). The polymorphism loci of *CYP2D6∗10*, *AGTR1* (*1166A* > *C*), and *NPPA* (*2238T* > *C*) did not conform to gene balance (*P* < 0.05) (Table 3). Univariate analysis revealed that the *CC* genotype of *ADRB1* (*1165G* > *C*) was strongly associated with a higher stroke risk than the *GC* and *CC* genotypes (*χ*^2^ = 8.659, *P* < 0.05) (Table 3). This association remained significant after multivariate unconditional logistic regression analysis was performed adjusting for age, sex, BMI, type of usual residence, education, marital status, control of SBP, control of DBP, presence of disease, family history, and antihypertensive drug history. These observations revealed that the risk of stroke was 1.184 times higher in *CC* carriers with the dominant gene than in *GC* + *GG* genotype patients (95% confidence interval (CI): 1.026–1.365, *P* < 0.05) (Table 4). No association was observed between *CYP2D6∗10*,*CYP2C9∗3*, *AGTR1* (*1166A* > *C*), *ACE* (*I*/*D*), *CYP3A5∗3*, and *NPPA* (*2238T* > *C*) polymorphisms and stroke (Tables 3 and 4).

### 3.3. Subgroup Analysis of the Relationship between Antihypertensive Drug-Related Genes and Stroke in Hypertensive Patients

**Figure 2:**
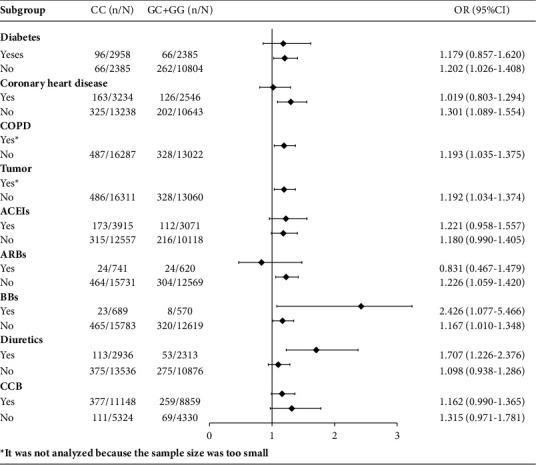
Forest plot of the association between *ADRB1* gene and stroke occurrence in different subgroups.

To further understand the specific association of *ADRB1* (*CC vs*. *GC* + *GG*) with stroke in different subpopulations, we conducted regression analysis by dividing the study subjects into different subgroups according to the presence of diabetes mellitus, presence of coronary heart disease, presence of COPD, presence of tumor, and whether they were taking BBs, ARBs, ACEIs, CCBs, and diuretics. The results revealed that *CC* (*vs GC* + *GG*) of the *ADRB1* (*1165G* > *C*) gene was associated with stroke occurrence in nondiabetic and noncoronary populations of hypertensive patients (OR: 1.20, 95% CI: 1.03–1.41; OR: 1.30, 95% CI: 1.09–1.56), in hypertensive patients who took BBs (OR: 2.43, 95% CI: 1.08–5.47; OR: 1.17, 95% CI: 1.010–1.348) or diuretics (OR: 1.707, 95% CI: 1.226–2.376) (Figure 2). No correlation was observed between *CYP2D6∗10*, *CYP2C9∗3*, *AGTR1* (*1166A* > *C*), *ACE* (*I*/*D*), *NPPA* (*2238T* > *C*), *CYP3A5∗3* genes polymorphism and stroke in hypertensive patients in subgroup analysis. For specific results, refer to the supplementary documents (Figures S1–S6).

## 4. Discussion

This study revealed that the prevalence of stroke in the hypertensive population in Changsha County of Hunan Province was 2.75%, which was significantly lower than the 10.04% reported in hospitalized hypertensive patients in the Anhui Province [31]. The difference may be a result of differences in the study population, with the hospitalized population having more difficulty in controlling BP and higher-grade hypertension than the community population. Furthermore, the BMI and age were lower in the present study than in the previous one. The prevalence of stroke in male patients was higher than that in female patients possibly because female patients had better hypertension control and a lower incidence of stroke than male patients [9].

Our study showed that the mutation frequencies of antihypertensive drug-related genes varied from 5.71% to 74.43%. The mutation rate of the *ADRB1* (*1165G* > *C*) gene (74.43%) was slightly higher than that in Chinese patients with essential hypertension reported previously (59.8%) [32], while the mutation rate of *CYP3A5∗3* (69.58%) was slightly lower than that reported previously (79.5%) [33]. The difference may be explained by the fact that the present study recruited hypertensive patients in the community, while the previous research subjects were hospitalized hypertensive patients [32] or healthy individuals [33]. The frequency of *CYP2D6∗10*, *CYP2C9∗3*, and *AGTR1* (*1166A* > *C*) was generally consistent with that reported for Chinese hypertensive patients in previous studies [21, 34]. In addition, we observed that *CYP2D6∗10*, *AGTR1* (*1166A* > *C*), and *NPPA* (*2238T* > *C*) genes do not conform to the Hardy–Weinberg equilibrium test. Another study conducted in Chinese Han hypertensive patients also found that *CYP2D6∗10* was not in HWE [16]; this might be because *CYP2D6∗10*, *AGTR1* (*1166A* > *C*), and *NPPA* (*2238T* > *C*) genes were associated with the development of hypertension [23, 35, 36]. Studies revealed that alleles associated with disease etiology often deviate from the expected allele or genotype frequency [37].

Our results indicated that the *CC* genotype of *ADRB1* (*1165G* > *C*) is a risk factor for stroke in hypertensive patients, and the risk of stroke was 1.184 times higher for the *CC* genotype than for the *GC* + *GG* genotype (95% CI: 1.026–1.365). Studies had established that *ADRB1* (*1165G* > *C*) dominant allele *C* was a risk factor for hypertension [38–41]. It is reasonable to assume that *CC* may also contribute to the risk of developing stroke in hypertensive patients. In addition, previous studies indicated that mutation of *ADRB1* (*1165G* > *C*) was associated with shorter sleep duration in humans and mice; carriers of the mutation sleep two hours less per day on average than nonmutation carriers [42], and shorter sleep duration was an independent risk factor for future stroke events in hypertensive patients [43]. Therefore, *ADRB1* (*1165G* > *C*) might also affect the occurrence of stroke by affecting sleep. Our study was inconsistent with the results of a study on *ADRB1* (*1165G* > *C*) polymorphism and ischemic stroke in North India, which recruited 224 patients and 224 age- and sex-matched controls and discovered no association between *ADRB1* (*GC* + *GG* vs. *CC*) and overall ischemic stroke [44]. The following reasons can explain the difference: (1) The difference of interested diseases: Our study recruited all patients diagnosed with ICD codes ranging from I60 to I64, while the Indian study only included patients with ischemic diseases (I63); (2) heterogeneity of design methods: This study is a cross-sectional study and the previous one is case-control study; and (3) the sample difference: This study had a larger number of subjects derived from the community, and the case control had a smaller sample size and was hospital-based. Although both studies had some bias, we believe that genes are generally associated with stroke.

To further analyze the relationship between *ADRB1* (*1165G* > *C*) polymorphism and stroke, we performed a subgroup analysis. The results indicated that *ADRB1* (*1165G* > *C*) polymorphism was significantly associated with stroke risk in patients with noncoronary heart disease and nondiabetic diseases, while not significantly associated with stroke risk in patients with coronary heart disease and diabetes. This finding suggests that hypertensive patients comorbid with coronary heart disease or diabetes may modify the association of *ADRB1* (*1165G* > *C*) polymorphisms with stroke. In patients only with elevated BP, the *CC* genotype was the main risk factor for stroke; in hypertensive patients with other comorbid conditions, many nongenetic factors related to coronary heart disease or diabetes, such as smoking, physical activities, and dietary factors [31, 45, 46], might affect stroke development and conceal the association of *ADRB1* (*1165G* > *C*) gene polymorphism with stroke. Similarly, a meta-analysis revealed no significant association of *ADRB1* (*1165G* > *C*) polymorphisms with cardiovascular events in patients with coronary artery disease [47].

The result of subgroup analysis revealed that polymorphisms in the *ADRB1* (*1165G* > *C*) gene were consistently associated with stroke in hypertensive patients regardless of BBs use. The OR values were 2.426 (95% CI: 1.077–5.466) and 1.167 (95% CI: 1.010–1.348) in hypertensive *CC* and *GC* + *GG* carriers, respectively. Numerous studies have demonstrated that the CC genotype is associated with a better antihypertensive response to metoprolol treatment in different racial populations of healthy volunteers and hypertensive patients [26, 48]. A recent study assessed the role of *ADRB1* (*1165G* > *C*) gene polymorphisms on BBs response in a population with heart failure, revealing that the patients with the CC genotype seemed to receive the most benefit from a high BBs dose. However, our study showed the opposite result. The discrepancy could be explained by the following reasons:

1. BBs are the first-line pharmacological treatment for hypertensive patient comorbid with other conditions, such as heart failure and coronary disease, but not the first-line treatment for simple blood pressure-elevated patients. Therefore, for those simple blood pressure elevated patients with the *CC* genotype, BBs treatment was prescribed until “trial and error” treatment failed or developed with other conditions, the benefit from BBs prescription of CC genotype patients may have been delayed, and lead to a higher risk of stroke.
2. Although we excluded the data of poststroke drug usage, the patients may have experienced a prolonged period of uncontrolled BP before the stroke, and during this period, BBs may have been used. A study showed that there may be a higher risk of stroke due to greater variability in BP when treated with BBs [49], increasing the risk among patients with BBs. Our results indicated that *ADRB1* (*1165G* > *C*) was not associated with stroke occurrence in hypertensive patients without diuretics, and *ADRB1* (*1165G* > *C*) was associated with stroke risk in hypertensive patients with diuretics, possibly because the patients without diuretics were those with a shorter course of the disease and a lower BP level. The antihypertensive effect of diuretics was relatively weak when used as antihypertensive drugs in hypertensive patients for a long time [50]. Therefore, long-term use of diuretics may lead to poor BP control and increase the risk of stroke.

We did not establish an association of *CYP2D6∗10*, *CYP2C9∗3*, *AGTR1* (*1166A* > *C*), *ACE* (*I*/*D*), *CYP3A5∗3*, and *NPPA* (*2238T* > *C*) gene polymorphisms with stroke susceptibility. The result of a study conducted in India was consistent with our observations, in that no significant association of *ACE* (*I*/*D*) gene polymorphisms with ischemic stroke exists in hypertensive patients [51]. The secondary *C* allele of the *NPPA* (*2238T* > *C*) gene is associated with a higher risk of stroke [52]; however, no association between the *NPPA* (*2238T* > *C*) gene and stroke susceptibility was detected in other studies [19]. Regarding the *AGTR1* (*1166A* > *C*) gene, studies revealed that its *C* allele was associated with increased BP in hypertensive patients [35]; currently, there are no studies on its relationship with stroke in hypertensive patients. Therefore, the association of *CYP2D6∗10*, *CYP2C9∗3*, *AGTR1* (*1166A* > *C*), *ACE* (*I*/*D*), *CYP3A5∗3*, and *NPPA* (*2238T* > *C*) gene polymorphisms with stroke in hypertensive patients needs further studies.

This study has some limitations. First, stroke is a disease with complex pathogenesis; hemorrhagic stroke and ischemic stroke have different mechanisms, conditions, and prognoses. The data used in this study could not distinguish between ischemic and hemorrhagic stroke. Therefore, the study could not analyze the differences between these two types of stroke. Second, all the participants were Han people, and there may be genetic variations among different ethnic groups. In future studies, we will verify the results of this study with data from other groups. Third, the stroke cases identified in the hypertension management system were mainly prevalent cases; therefore, the study was inclined to generate a Neiman bias. As we explained in the previous paragraph, although we excluded the poststroke data, the patients may have experienced a long period of uncontrolled BP before the stroke, and during this period, medications and lifestyles may have changed, leading to a bias. Therefore, new cases should be selected, and more accurate exposure assessment methods are needed in future studies. However, our study systematically investigated the association of antihypertensive drug-related gene polymorphisms and the occurrence of stroke in hypertensive patients by including a large number of participants to obtain reliable data through a hypertension management system and adjusting for possible nongenetic factors that may influence the occurrence of stroke, such as sex, age, marital status, education level, BMI, family history, and antihypertensive medications.

## Figures and Tables

**Table 1:** Demographic characteristics and associated risk factors of patients with stroke and healthy controls.

Variables	Categories	Controls	Stroke patients	*P*
Sex	Male	11496 (39.9)	406 (49.8)	<0.001
	Female	17349 (60.1)	410 (50.2)	

Age (y)		64.63 ± 9.89	67.20 ± 8.60	<0.001

BMI (kg/m^2^)		23.88 ± 3.19	23.36 ± 3.10	<0.001

Type of usual residence	Country	27256 (94.5)	769 (94.2)	0.757
	City	1589 (5.5)	47 (5.8)	

Marital status	Unmarried	583 (2.0)	16 (2.0)	0.409
	Married	25352 (87.9)	706 (86.5)	
	Divorced or widowed	2910 (10.1)	94 (11.5)	

Educational background	Primary school and below	15573 (54.0)	491 (60.2)	0.002
	Junior high school	11234 (38.9)	273 (33.4)	
	High school and above	2038 (7.1)	52 (6.4)	

Control of SBP	No	16490 (57.2)	488 (59.8)	0.133
	Yes	12355 (42.8)	328 (40.2)	

Control of DBP	No	11695 (40.5)	308 (37.7)	0.108
	Yes	17150 (59.5)	508 (62.3)	

Diabetes	No	23664 (82.0)	654 (80.1)	0.166
	Yes	5181 (18.0)	162 (19.9)	

Coronary heart disease	No	23354 (81.0)	527 (64.6)	<0.001
	Yes	5491 (19.0)	289 (35.4)	

COPD	No	28494 (98.8)	815 (99.9)	0.004
	Yes	351 (1.2)	1 (0.1)	

Tumor	No	28557 (99.0)	814 (99.8)	0.031
	Yes	288 (1.0)	2 (0.2)	

Family history of hypertension	No	25416 (88.1)	692 (84.8)	0.004
	Yes	3429 (11.9)	124 (15.2)	

Family history of stroke	No	28494 (98.8)	796 (97.5)	0.002
	Yes	351 (1.2)	20 (2.5)	

Family history of diabetes	No	28399 (98.5)	802 (98.3)	0.699
	Yes	446 (1.5)	14 (1.7)	

ACEIs	No	22144 (76.8)	531 (65.1)	<0.001
	Yes	6701 (23.2)	285 (34.9)	

ARBs	No	27532 (95.4)	768 (94.1)	0.073
	Yes	1313 (4.6)	48 (5.9)	

BBs	No	27617 (95.7)	785 (96.2)	0.522
	Yes	1228 (4.3)	31 (3.8)	

Diuretics	No	23762 (82.4)	650 (79.7)	0.045
	Yes	5083 (17.6)	166 (20.3)	

CCBs	No	9474 (32.8)	180 (22.1)	<0.001
	Yes	19371 (67.2)	636 (77.9)	

COPD, chronic obstructive pulmonary disease; ACEI, angiotensin-converting enzyme inhibitor; ARBs, angiotensin-II receptor antagonists; BBs, beta receptor blockers; CCBs, calcium channel blockers.

**Table 2:** Genotypes and allele frequency distribution in hypertensive population.

Gene loci	Genotype (%)			Allele (%)	
	WW	WM	MM	W	M
*ADRB1* (*1165G* > *C*)	1971 (6.6)	11218 (37.8)	16472 (55.5)	15160 (25.6)	44162 (74.4)
*CYP2D6∗ 10*	6255 (21.1)	12855 (43.3)	10551 (35.6)	25365 (42.8)	33957 (57.2)
*CYP2C9∗ 3*	27204 (91.7)	2387 (8.0)	70 (0.2)	56795 (95.7)	2527 (4.3)
*AGTR1* (*1166A* > *C*)	26458 (89.2)	3021 (10.2)	182 (0.6)	55937 (94.3)	3385 (5.7)
*ACE* (*I*/*D*)	13959 (47.1)	12646 (42.6)	3056 (10.3)	40564 (68.4)	18758 (31.6)
*CYP3A5∗ 3*	2783 (9.4)	12473 (42.1)	14405 (48.6)	18039 (30.4)	41283 (69.6)
*NPPA* (*2238T* > *C*)	29091 (98.1)	443 (1.5)	127 (0.4)	58625 (98.8)	697 (1.2)

WW, wild-type homozygotes; WM, heterozygous mutation; MM, homozygous mutation; W, wild type; M. mutant.

**Table 3:** Distribution of polymorphisms in genes related to antihypertensive drugs.

Gene loci	Genotype	Controls		Stroke patients		*χ* ^2^	*P*
		*n*	%	*n*	%		
*ADRB1* (*1165G* > *C*)	*CC*	15984	55.4	488	59.8	8.884	0.012
	*GC*	10950	38.0	268	32.8		
	*GG*	1911	6.6	60	7.4		

HWE-P		>0.05					

*CYP2D6∗ 10*	*∗1*/*∗1*	6085	21.1	170	20.8	3.097	0.213
	*∗1*/*∗10*	12522	43.4	333	40.8		
	*∗10*/*∗10*	10238	35.5	313	38.4		

HWE-P		<0.05					

*CYP2C9∗ 3*	*∗1*/*∗1*	26452	91.7	752	92.2	2.042	0.360
	*∗1*/*∗3*	2323	8.1	64	7.8		
	*∗3*/*∗3*	70	0.2	0	0.0		

HWE-P		>0.05					

*AGTR1* (*1166A* > *C*)	*1166A*/*A*	25721	89.2	737	90.3	1.585	0.453
	*1166A*/*C*	2948	10.2	73	8.9		
	*1166C*/*C*	176	0.6	6	0.7		

HWE-P		<0.05					

*ACE* (*I*/*D*)	*II*	13564	47.0	395	48.4	0.666	0.717
	*ID*	12305	42.7	341	41.8		
	*DD*	2976	10.3	80	9.8		

HWE-P		>0.05					

*CYP3A5∗ 3*	*∗1*/*∗1*	2700	9.4	83	10.2	1.537	0.464
	*∗1*/*∗3*	12120	42.0	353	43.3		
	*∗3*/*∗3*	14025	48.6	380	46.6		

HWE-P		>0.05					

*NPPA* (*2238T* > *C*)	*2238T*/*T*	28291	98.1	800	98.0	0.128	0.938
	*2238T*/*C*	430	1.5	13	1.6		
	*2238C*/*C*	124	0.4	3	0.4		

HWE-P		<0.05					

**Table 4:** Association between antihypertensive drug-related genes polymorphism and stroke occurrence in hypertensive patients (controls = 28846 and stroke = 816).

Gene loci	Crude OR (95% CI)	Adjusted OR^‡^ (95% CI)	Adjusted OR^§^ (95% CI)
*ADRB1* (*1165G* > *C*)			
*CC*	1.197 (1.039–1.380)^*∗*^	1.186 (1.029–1.368)^*∗*^	1.184 (1.026–1.365)^*∗*^
*GC* + *GG*	1^†^	1^†^	1^†^
*GG*	1.119 (0.857–1.461)	1.186 (1.029–1.370)	1.099 (0.840–1.438)
*GC* + *CC*	1^†^	1^†^	1^†^

*CYP2D6∗10*			
*∗10*/*∗10*	1.131 (0.980–1.305)	1.119 (0.969–1.292)	1.115 (0.966–1.288)
*∗1*/*∗1*+*∗1*/*∗10*	1^†^	1^†^	1^†^
*∗1*/*∗1*	0.984 (0.829–1.168)	0.982 (0.827–1.166)	0.981 (0.825–1.166)
*∗1*/*∗10*+*∗10*/*∗10*	1^†^	1^†^	1^†^

*CYP2C9∗3*			
*∗1*/*∗1*	1.063 (0.821–1.377)	1.033 (0.796–1.339)	1.021 (0.787–1.324)
*∗1*/*∗3*+*∗3*/*∗3*	1^†^	1^†^	1^†^
*∗3*/*∗3*	Not available	Not available	Not available
*∗1*/*∗3*+*∗1*/*∗1*	1^†^	1^†^	1^†^

*AGTR1* (*1166A* > *C*)			
*1166A*/*A*	1.133 (0.896–1.433)	1.115 (0.881–1.412)	1.122 (0.885–1.421)
*1166A*/*C*+*1166C*/*C*	1^†^	1^†^	1^†^
*1166C*/*C*	1.207 (0.533–2.730)	1.293 (0.569–2.937)	1.279 (0.562–2.911)
*1166A*/*C*+*1166A*/*A*	1^†^	1^†^	1^†^

*ACE* (*I*/*D*)			
*II*	1.057 (0.920–1.215)	1.077 (0.936–1.239)	1.078 (0.937–1.240)
*ID* + *DD*	1^†^	1^†^	1^†^
*DD*	0.945 (0.748–1.194)	0.921 (0.728–1.165)	0.923 (0.729–1.168)
*ID* + *II*	1^†^	1^†^	1^†^

*CYP3A5∗3*			
*∗3*/*∗3*	0.921 (0.801–1.059)	0.936 (0.814–1.077)	0.938 (0.815–1.079)
*∗1*/*∗3*+*∗1*/*∗1*	1^†^	1^†^	1^†^
*∗1*/*∗1*	1.096 (0.871–1.381)	1.072 (0.850–1.351)	1.080 (0.856–1.362)
*∗1*/*∗3*+*∗3*/*∗3*	1^†^	1^†^	1^†^

*NPPA* (*2238T* > *C*)			
*TT*	0.979 (0.593–1.617)	0.973 (0.587–1.612)	0.962 (0.580–1.595)
*TC* + *CC*	1^†^	1^†^	1^†^
*CC*	0.855 (0.271–2.692)	0.942 (0.296–2.994)	0.946 (0.297–3.010)
*TC* + *TT*	1^†^	1^†^	1^†^

^*∗*^ *P* < 0.05. ^†^Reference. ^‡^Adjusted for age, sex, BMI, type of usual residence, education, marital status, control of SBP, control of DBP, presence of diabetes, presence of coronary heart disease, presence of COPD, presence of tumor, and family history related to hypertension, stroke, diabetes as covariates. ^§^Adjusted for age, sex, BMI, type of usual residence, education, marital status, control of SBP, control of DBP, presence of diabetes, presence of coronary heart disease, presence of COPD, presence of tumor, family history related to hypertension, stroke, diabetes, and whether taking BBs, ACEIs, ARBs, CCBs, and diuretics as covariates.

## Data Availability

The data used to support the findings of this study are available from the corresponding author upon request.

## Supplementary Materials

Supplementary Materials Supplementary Table 1: rs number of genes and interested genotype. Supplementary Figure 1: forest plot of the association between *CYP2D6∗10* gene and stroke occurrence in different subgroups. Supplementary Figure 2: forest plot of the association between *CYP2C9∗3* gene and stroke occurrence in different subgroups. Supplementary Figure 3: forest plot of the association between *AGTR1* (*1166A* > *C*) gene and stroke occurrence in different subgroups. Supplementary Figure 4: forest plot of the association between *ACE* (*I*/*D*) gene and stroke occurrence in different subgroups. Supplementary Figure 5: forest plot of the association between *CYP3A5∗3* gene and stroke occurrence in different subgroups. Supplementary Figure 6: forest plot of the association between *NPPA* (*2238T* > *C*) gene and stroke occurrence in different subgroups.
