# Supplementary material for: Association of Antihypertensive Drug-Related Gene Polymorphisms with Stroke in the Chinese Hypertensive Population
Source: Int J Hypertens. 2024 Jan 23;2024:5528787. doi: 10.1155/2024/5528787 (PMC10827366; doi:10.1155/2024/5528787)
Supplement: Supplementary Materials — Supplementary Table 1: rs number of genes and interested genotype. Supplementary Figure 1: forest plot of the association between CYP2D6∗10 gene and stroke occurrence in different subgroups. Supplementary Figure 2: forest plot of the association between CYP2C9∗3 gene and stroke occurrence in different subgroups. Supplementary Figure 3: forest plot of the association between AGTR1 (1166A > C) gene and stroke occurrence in different subgroups. Supplementary Figure 4: forest plot of the association between ACE (I/D) gene and stroke occurrence in different subgroups. Supplementary Figure 5: forest plot of the association between CYP3A5∗3 gene and stroke occurrence in different subgroups. Supplementary Figure 6: forest plot of the association between NPPA (2238T > C) gene and stroke occurrence in different subgroups. [file 5528787.f1.docx]

Supplementary Material

*Table*

Table S1 RS Number of Gene and Interested Genotype

| Gene (polymorphism) | rs Number | Interested Genotype | W | M |  |
| --- | --- | --- | --- | --- | --- |
| *ADRB1(1165 G>C)* | rs1801253 | *CC*, *GG* | *G* | *C* |  |
| *AGTR1 (1166A>C)* | rs5186 | *CC*, *AA* | *A* | *C* |  |
| *ACE (Insertion/Deletion)* | rs4646994 | *DD*, *II* | *I* | *D* |  |
| *NPPA (2238T>C)* | rs5063 | *CC, TT* | *T* | *C* |  |
| *CYP2D6* (**1* - **171*)† | | **1*, **10* | **1* | **10* |  |
| *CYP2C9*(**1* - **85*)† | | **1*, **3* | **1* | **3* |  |
| *CYP3A5*(**1* - **9*)† | | **1*, **3* | **1* | **3* |  |

† No rs Number. W, Wild type; M. mutant type.

*Figures*


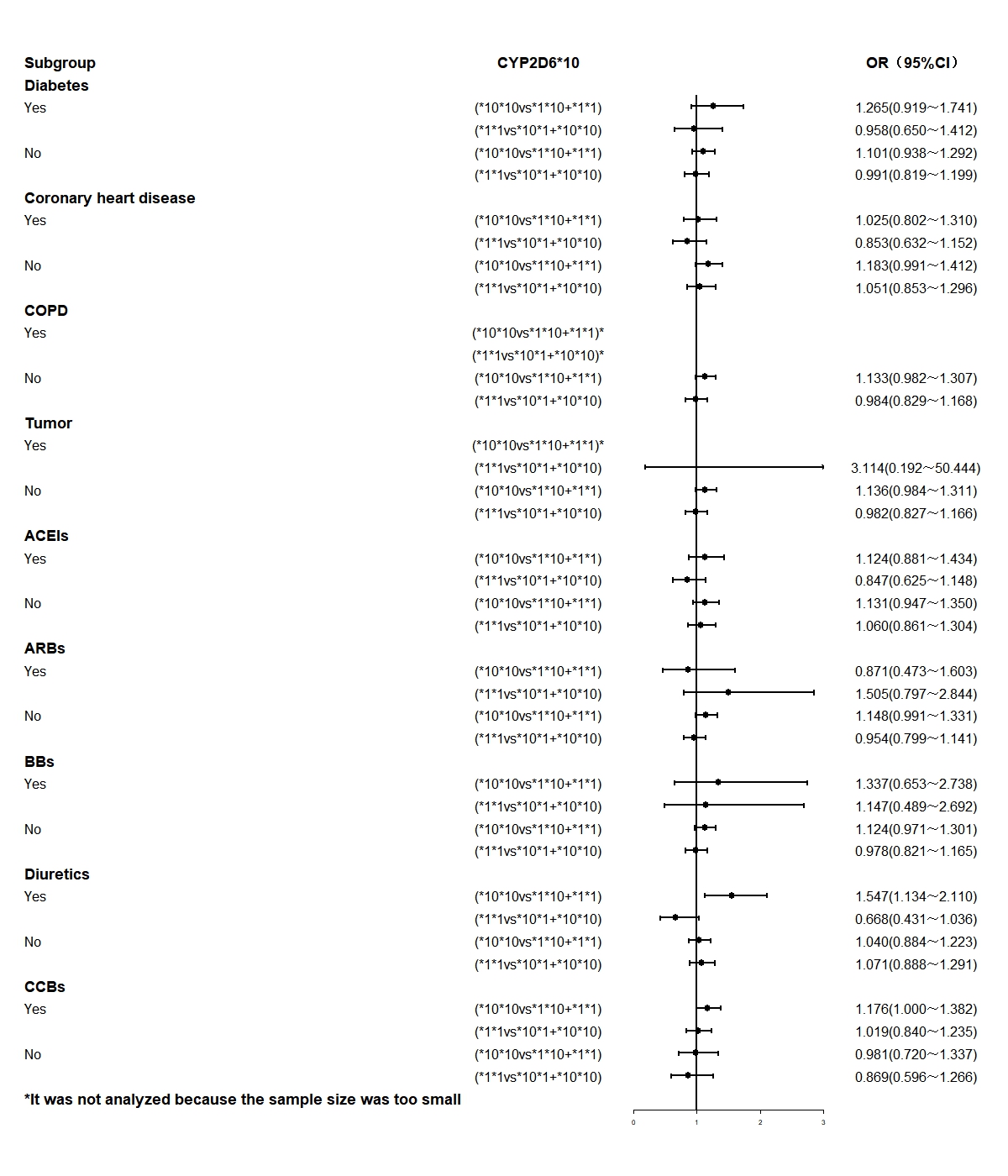


Figure S1 Forest plot of the association between *CYP2D6*10* gene and stroke occurrence in different subgroups

No association was observed between *CYP2D6*10* polymorphisms and stroke.


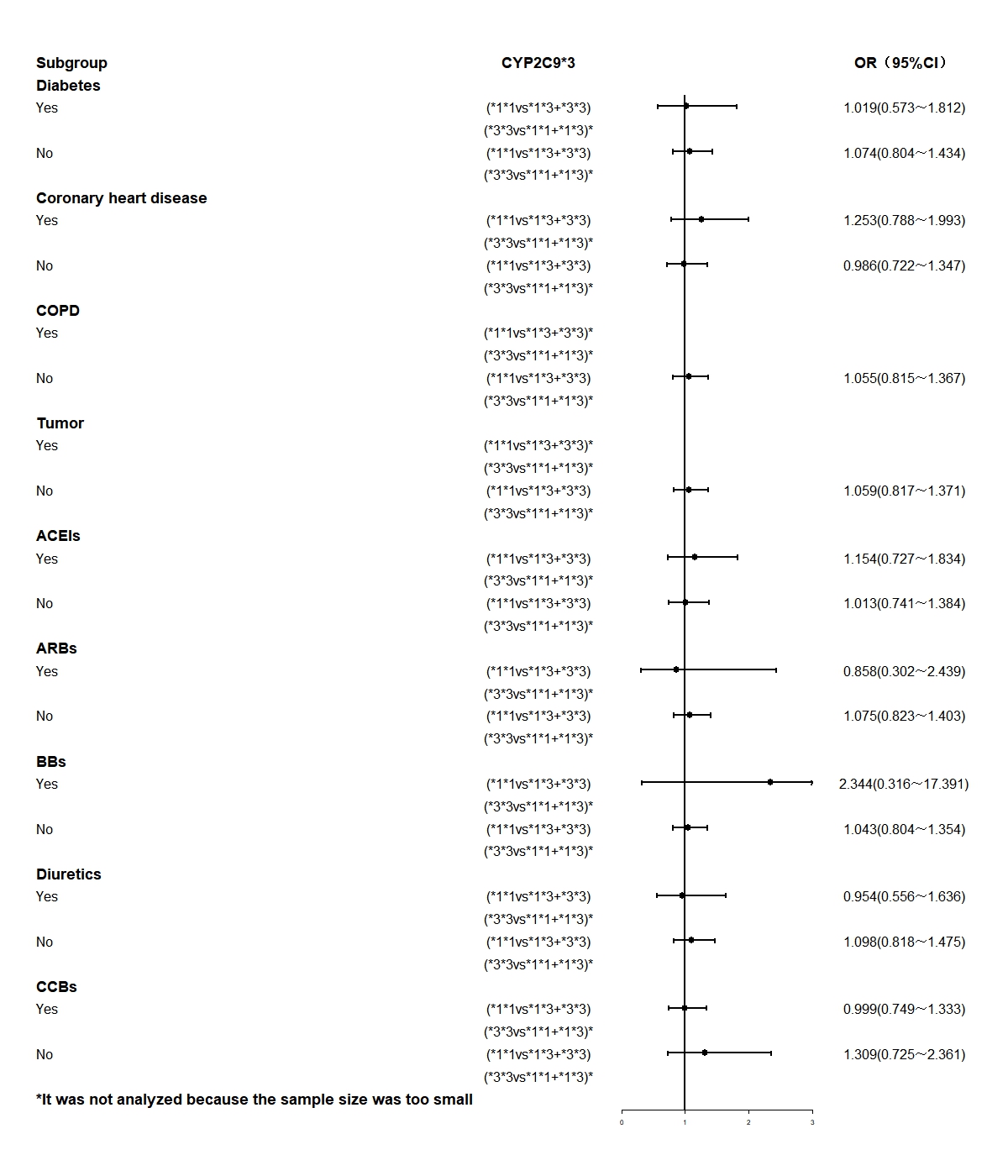


Figure S2 Forest plot of the association between *CYP2C9*3* gene and stroke occurrence in different subgroups

No association was observed between *CYP2C9*3* polymorphisms and stroke.


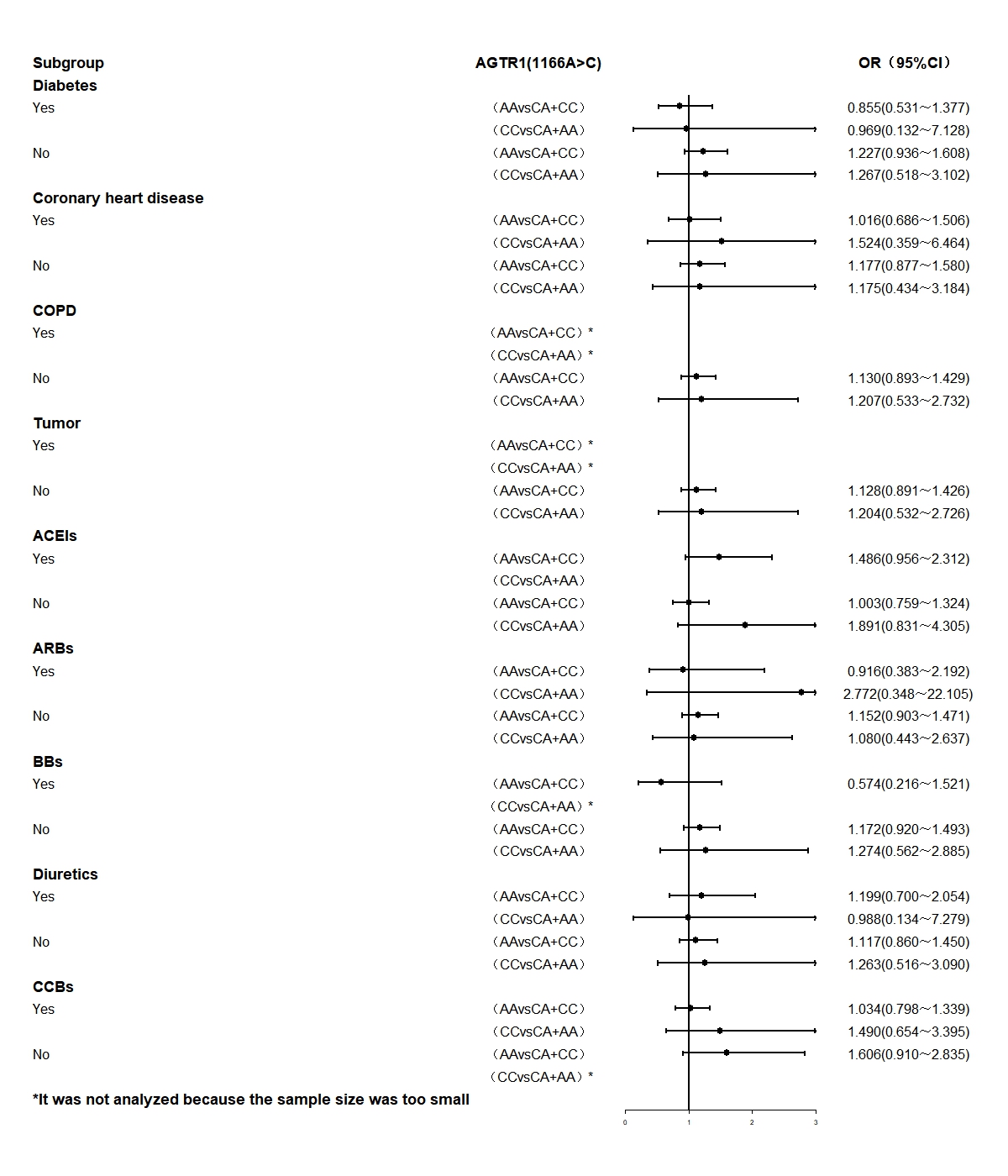
Figure S3 Forest plot of the association between *AGTR1(1166A>C)* gene and stroke occurrence in different subgroups

No association was observed between *AGTR1(1166A>C)* polymorphisms and stroke.


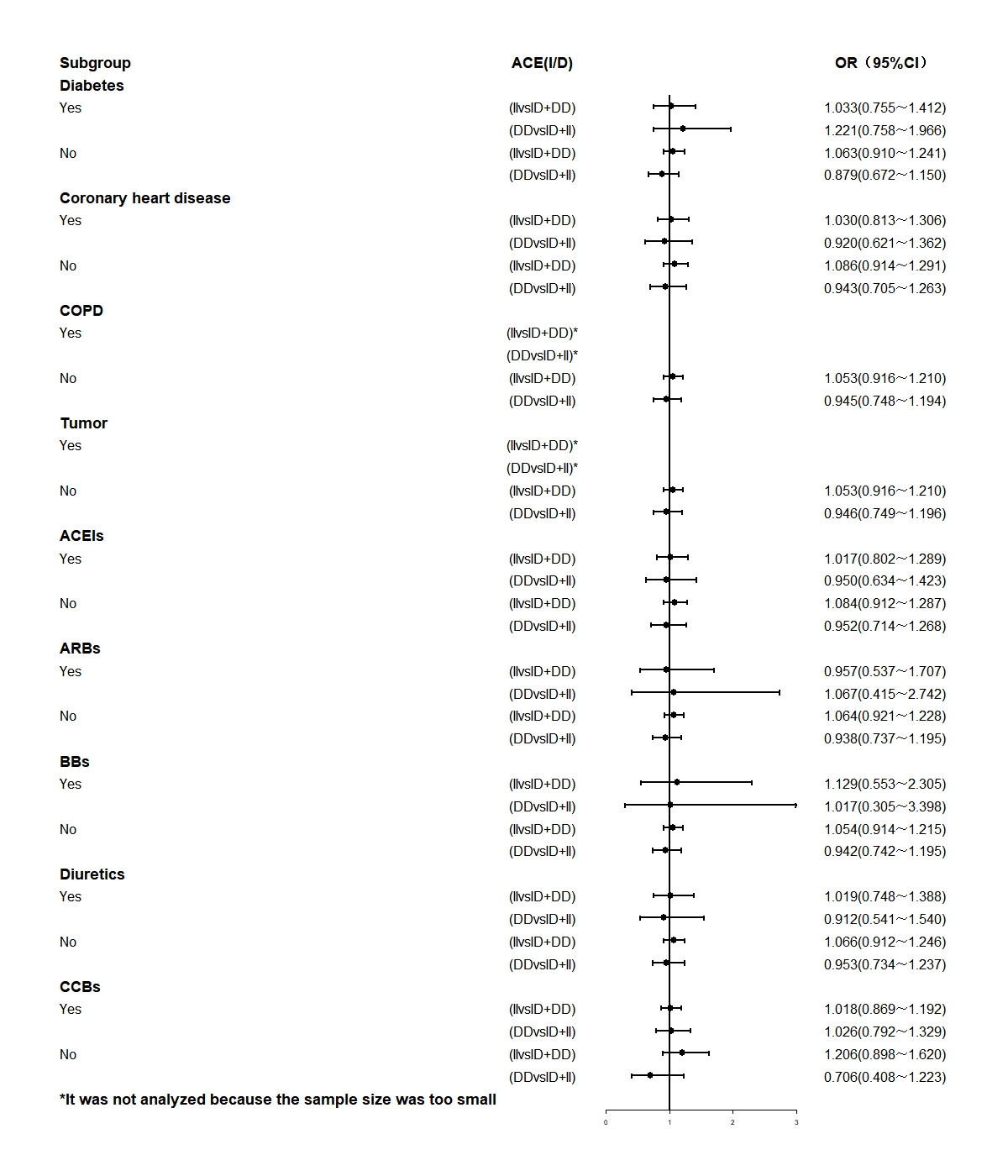
Figure S4 Forest plot of the association between *ACE(I/D)* gene and stroke occurrence in different subgroups

No association was observed between *ACE (I/D)* polymorphisms and stroke.


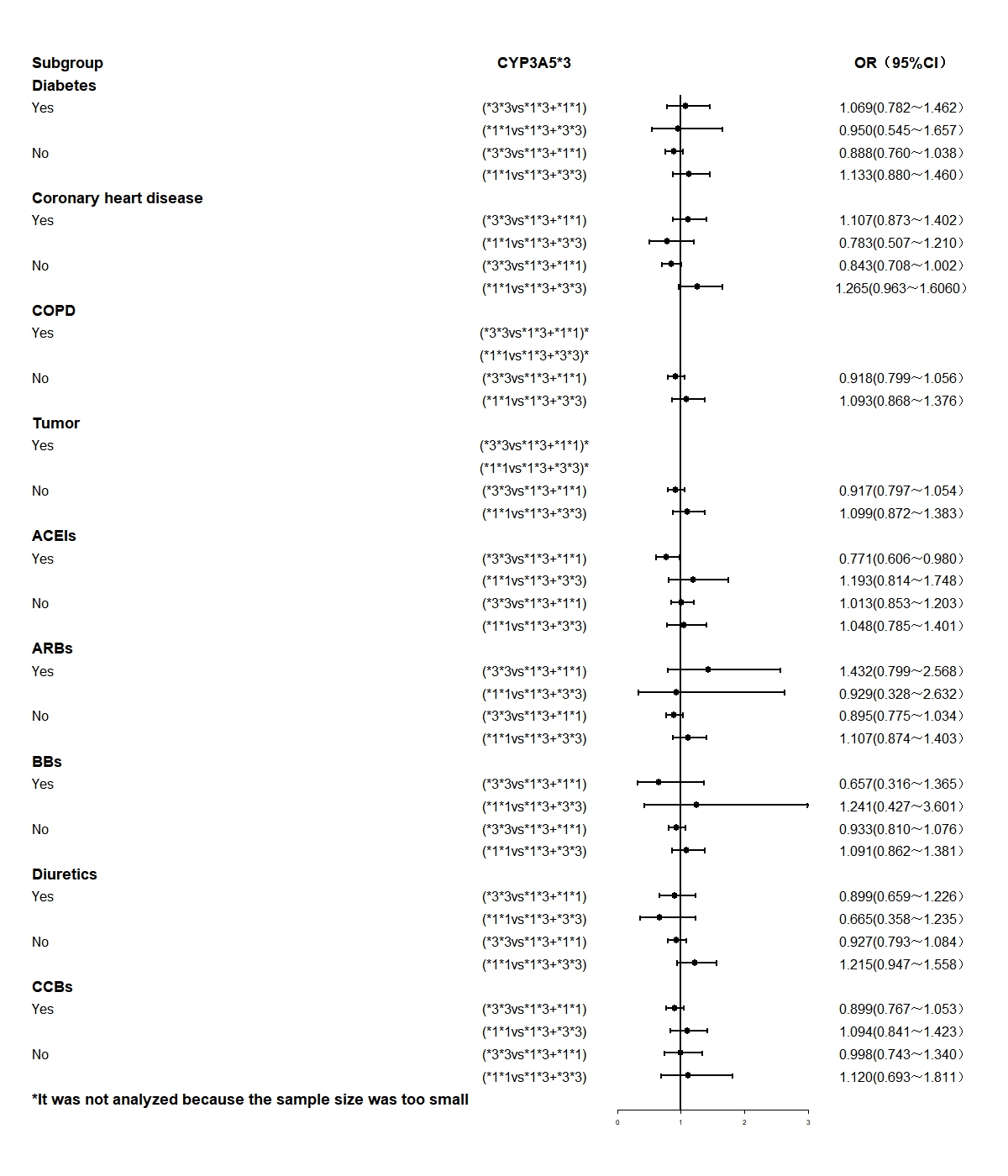
Figure S5 Forest plot of the association between *CYP3A5*3* gene and stroke occurrence in different subgroups

No association was observed between *CYP3A5*3* polymorphisms and stroke.


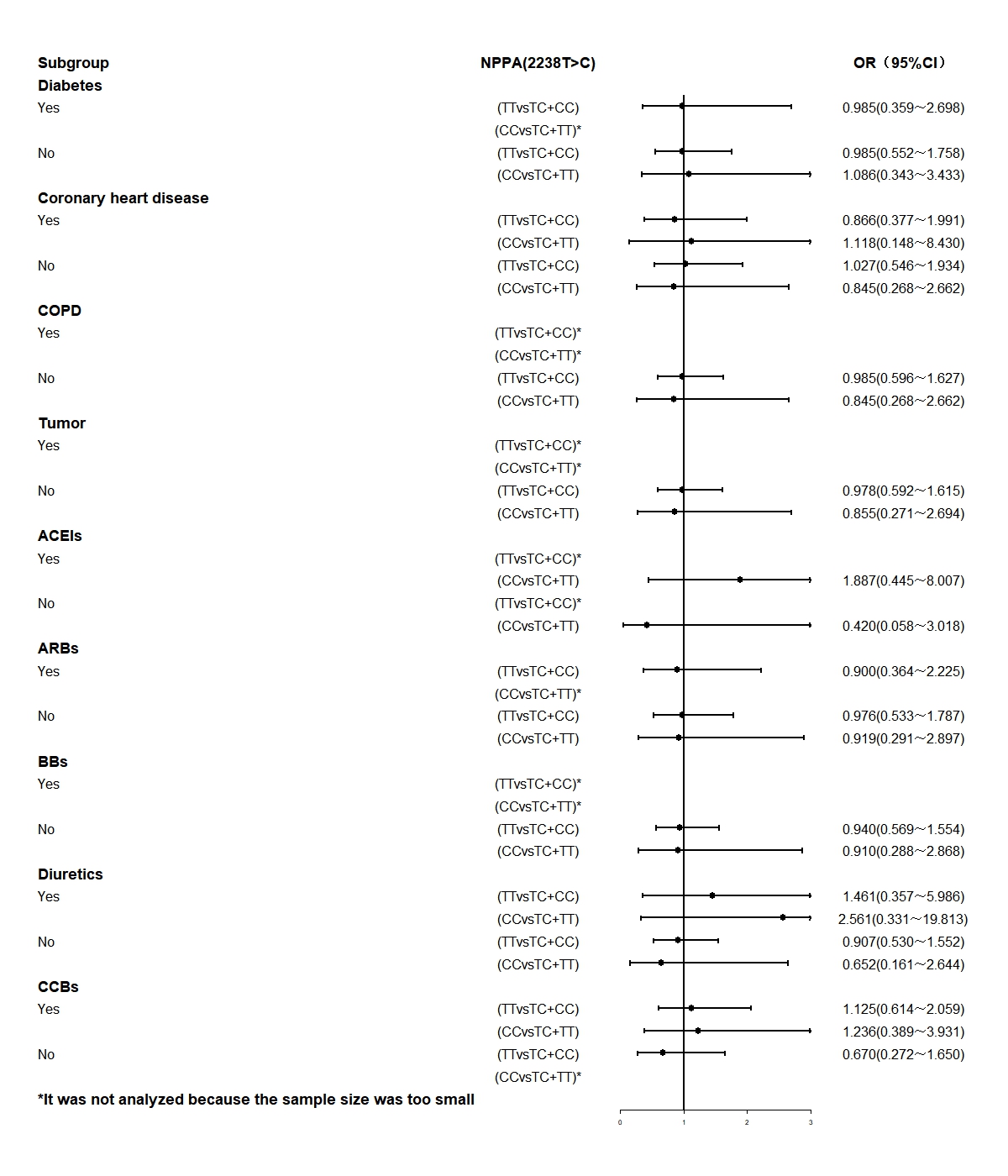
Figure S6 Forest plot of the association between *NPPA(2238T>C)* gene and stroke occurrence in different subgroups

No association was observed between *NPPA (2238T>C)* polymorphisms and stroke.
